# Modafinil treatment modulates functional connectivity in stroke survivors with severe fatigue

**DOI:** 10.1038/s41598-019-46149-0

**Published:** 2019-07-04

**Authors:** Milanka M. Visser, Peter Goodin, Mark W. Parsons, Thomas Lillicrap, Neil J. Spratt, Christopher R. Levi, Andrew Bivard

**Affiliations:** 1 School of Medicine and Public Health, Faculty of Health and Medicine, University of Newcastle, University Drive, Callaghan, 2308 New South Wales, Australia; 2 Department of Neurology, Royal Melbourne Hospital, University of Melbourne, Melbourne, Australia; 3 Department of Neurology, John Hunter Hospital, University of Newcastle, Newcastle, Australia; 4 School of Biomedical Sciences and Pharmacy, Faculty of Health and Medicine, University of Newcastle, Newcastle, Australia

**Keywords:** Stroke, Fatigue

## Abstract

Post-stroke fatigue has a significant impact on stroke survivors’ mental and physical well-being. Our recent clinical trial showed significant reduction of post-stroke fatigue with modafinil treatment, however functional connectivity changes in response to modafinil have not yet been explored in stroke survivors with post-stroke fatigue. Twenty-eight participants (multidimensional fatigue inventory-20 ≥ 60) had MRI scans at baseline, and during modafinil and placebo treatment. Resting-state functional MRI data were obtained, and independent component analysis was used to extract functional networks. Resting-state functional connectivity (rsFC) was examined between baseline, modafinil and placebo treatment using permutation testing with threshold-free cluster enhancement. Overall twenty-eight participants (mean age: 62 ± 14.3, mean baseline MFI-20: 72.3 ± 9.24) were included. During modafinil treatment, increased rsFC was observed in the right hippocampus (p = 0.004, 11 voxels) compared to placebo. This coincided with lower rsFC in the left frontoparietal (inferior parietal lobule, p = 0.023, 13 voxels), somatosensory (primary somatosensory cortex; p = 0.009, 32 voxels) and mesolimbic network (temporal pole, p = 0.016, 35 voxels). In conclusion, modafinil treatment induces significant changes in rsFC in post-stroke fatigue. This modulation of rsFC may relate to a reduction of post-stroke fatigue; however, the relationship between sensory processing, neurotransmitter expression and fatigue requires further exploration.

## Introduction

Fatigue is a highly prevalent post-stroke symptom with 60–70% of patients reporting fatigue in the first days after stroke^1^. Although symptom resolution is observed in many stroke survivors, around 40% of patients experience persistent fatigue for months or years^2^. Excessive tiredness limits the ability of stroke survivors to perform activities of daily living^3^, partake in social events^4^, and to return to work^5^. Post-stroke fatigue has a negative impact on rehabilitation, quality of life^6^, and is a predictor of mortality two years after stroke onset^7^.

The mechanisms underlying early and non-resolving chronic fatigue after stroke are poorly understood, and previous studies could not conclusively show a link between stroke-specific characteristics such as clinical stroke severity, stroke lesion location or lesion volume to the onset or prevalence of fatigue^8^. Therefore, it has been suggested that more general mechanisms may contribute to post-stroke fatigue such as decreased physical activity^9^, decreased cortical excitability^10^, or immune activity^11,12^, which decreases the availability of neurotransmitters such as dopamine and serotonin^13^.

Treatment of post-stroke fatigue is an ongoing challenge, as pharmacological treatments (e.g. fluoxetine) have failed to show positive results in the reduction of post-stroke fatigue^14^. Additionally, non-pharmacological therapies consisting of physical therapy and cognitive behavioural therapy are available; however, their efficacy has not been established in large, high-quality trials^14^. These non-pharmacological interventions may also not be viable for patients with severe fatigue, as the fatigue itself may interfere with their ability to participate in such a treatment^15^, indicating a need for pharmacological management of fatigue.

A previous small phase II trial of 200 mg of modafinil given daily for six weeks showed promising results by significantly reducing self-reported fatigue and increasing quality of life^16^. Modafinil is a weak dopamine-reuptake inhibitor that upregulates expression of orexin, a neuropeptide involved in wakefulness and attention. Depletion of orexin in the brain has been associated with narcolepsy and its associated bouts of excessive sleepiness^17^. Upregulation of expression of orexin in response to modafinil treatment is therefore thought to increase wakefulness, which is suggested to be beneficial in the management of fatigue.

Global changes in communication between brain areas can be assessed using resting-state functional connectivity (rsFC) analysis using functional MRI (fMRI). To investigate whether modafinil treatment and the reduction in post-stroke fatigue coincides with modulation of rsFC in the brain; we collected resting-state fMRI data in a group of patients with severe post stroke fatigue who were treated with modafinil during a clinical trial^18^. We investigated whether modafinil modulates rsFC in attention and cognition-related networks in patients with post-stroke fatigue. The primary analysis in this study was to compare functional connectivity within networks comparing modafinil treatment and placebo treatment. We hypothesized that modafinil would increase rsFC in cognition and attention-related networks, based on studies in healthy subjects^19,20^.

## Methods

**Figure 1:**
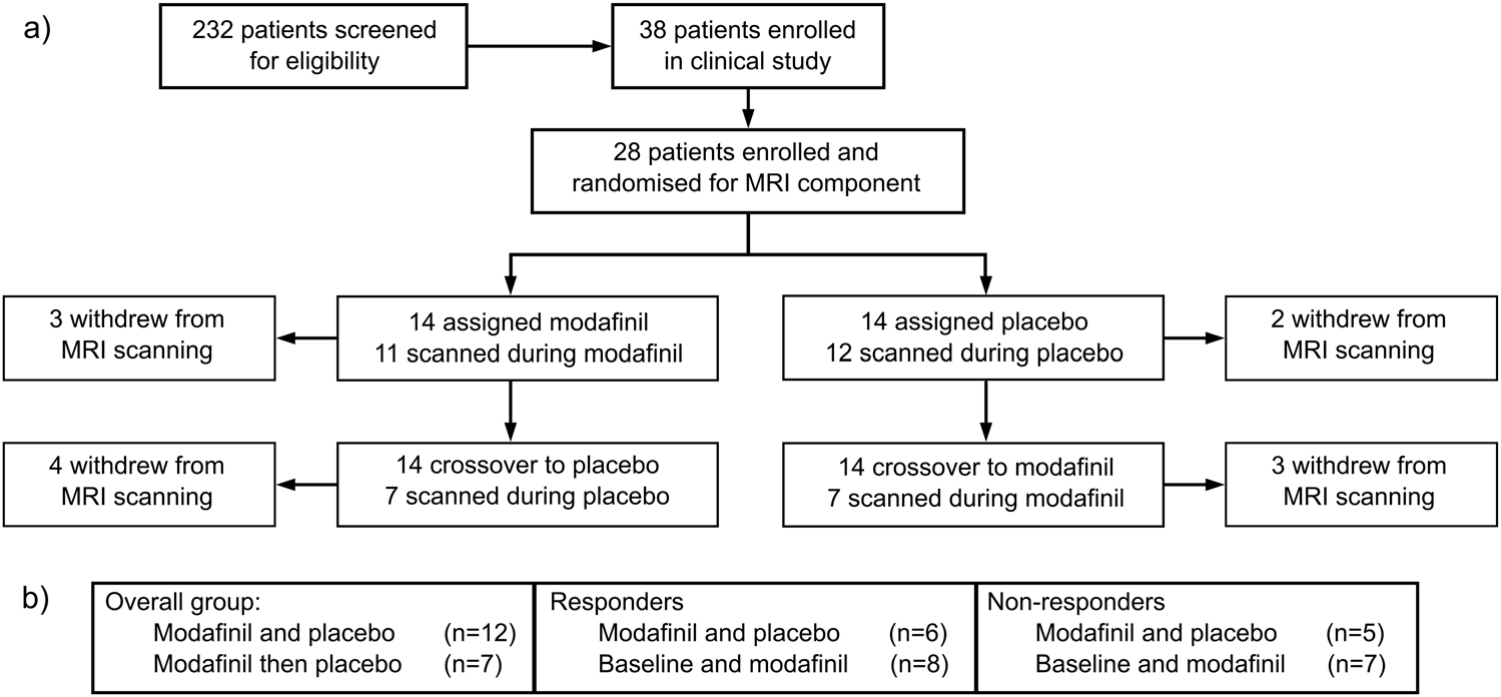
Flowchart of the MRI component of the modafinil in debilitating fatigue (MIDAS) trial. (**a**) After screening for eligibility, patients were enrolled to the study and randomized to either the modafinil treatment arm or the placebo treatment arm. The first six-week treatment period was followed by a one-week washout period after which crossover occurred in which patients received the alternative treatment to their allocation. (**b**) Number of included scans per analysis.

For this study, we accessed data from the Modafinil In Debilitating fatigue After Stroke (MIDAS) trial, a phase-II, single-centre, placebo-controlled, double-blind, randomized crossover trial^16^. The study protocol was registered with the Australian New Zealand Clinical Trials Registry (ACTRN12615000350527; date of registration: 17/04/2015)^18^. As part of the MIDAS trial, patients were randomized (1:1) to either modafinil (200 mg) or placebo for 6 weeks (daily via oral administration), and as part of the cross-over design, the study medication was swapped for another 6 weeks following a one-week washout period (Fig. 1). Clinical measures and magnetic resonance imaging (MRI) were captured prior to treatment (baseline), in the last week of the first 6-week treatment period and in the last week of the second treatment period. Inclusion criteria for MIDAS were patients of >18 years with a history of ischaemic stroke more than 3 months prior to consent, and a score of ≥60 on the multidimensional fatigue inventory (MFI-20) across all domains. Exclusion criteria were known contraindications to modafinil, presence of other known causes of fatigue and neuropsychiatric disease (e.g. pre-existing clinical depression and dementia) and sleep apnoea (if suspected by enrolling neurologist). Exclusion criteria for the MRI-specific component of the study were the presence of ferromagnetic bodies and claustrophobia. This study was approved by the Hunter New England Area Health District Human Research Ethics Committee. All participants provided written informed consent obtained in accordance with the Declaration of Helsinki.

### Study measurements

Clinical measures taken at all study time points included the multi-dimensional fatigue inventory (MFI-20), the Montreal Cognitive Assessment (MoCA), the Stroke Specific Quality of Life Scale (SSQoL), and the Depression, Anxiety and Stress Scales (DASS-42).

### Image acquisition

Participants underwent multi-modal MRI scanning sessions in a 3-Tesla Magnetom Prisma (Siemens Healthcare, Erlingen, Germany). During scanning sessions, a high-resolution T1-weighted anatomical scan (repetition time = 2300 ms, echo time = 1.69 ms, flip angle = 9°, 176 × 240 × 256 matrix size, 1 mm isotropic resolution, total acquisition time = 5 min) was obtained. Structural images were used for registration purposes and to create lesion masks needed to optimize registration. Additionally, rs-fMRI BOLD (blood oxygenation level dependent) T2*-weighted series were obtained with a gradient echo-planar-imaging (EPI) acquisition (250 volumes, repetition time = 2390 ms, echo time = 24.0 ms, flip angle = 90°, 88 × 88 × 47 matrix size, 3 mm isotropic resolution, total acquisition time = 10 min). During resting-state series acquisition, patients received instructions to lie still with eyes opened, and to not fall asleep. Total scanning time was approximately 30 minutes.

### Image preprocessing

Imaging data were preprocessed within the Nipype framework^21^. Resting-state series were visually checked for quality and the first 10 volumes of the time-series were deleted to remove any T1-attenuation effects. Volumes were consecutively de-spiked using 3dDespike from the Analysis of Functional NeuroImages (AFNI) program, slice-time corrected with respect to the middle slice using Statistical Parametric Mapping (SPM12, version 6906), and motion-corrected using the 24-parameter model^22^. Subsequently, the component-based noise correction (aCompCor) method was applied to remove signal from cerebrospinal fluid and white matter masks^23^. This was achieved by segmenting the structural images to create white matter, grey matter and cerebrospinal fluid masks and which were registered to the EPI images using SPM. The resting-state time-series were registered to the MNI-template using AntsRegistration package from the Advanced Normalization Tools (ANTs, version 1.9)^24^. To improve registration lesion masks (which were manually drawn on the structural scans) were included in the registration process. Then, registered resting-state series were band-pass filtered (0.08–0.1 Hz) using a Fourier filter and smoothed with a Gaussian kernel of 6 mm full-width at half-maximum (SPM).

To identify resting-state networks we performed independent component analysis using temporal concatenation of all included scans using melodic from the fMRI of the brain software library (FSL, version 5.0.9, Oxford, United Kingdom)^25^. Data were reduced to 8 independent components, which were subsequently supplied to dual regression in FSL to obtain participant-specific networks^26,27^. We selected the components corresponding to the networks-of-interest based on their visual concordance with previously defined resting-state networks^28–30^.

For the pooled analysis, scans were included in the modafinil treatment group irrespective of whether the patients received the active agent in treatment arm one or two, and similarly for the placebo group. We worked under the assumption that functional connectivity would return to the baseline state following the one week wash out period given that the half-life of modafinil is 10 to 12 hours. The imaging analysis required that data was available at least two time points. Given that not all participants completed MRI scanning at each time point (baseline, modafinil and placebo) imaging analysis was restricted to those that participants with available MRI scans.

### Imaging statistical analysis

Within-subject differences were assessed using FSL’s randomise with the maximum number of possible sign-flips (i.e. one-sample t-test equivalent of permutation) for each respective analysis (i.e. 2^n^). Family-wise errors where corrected using the threshold-free cluster enhancement and the significance level was set at p < 0.05. rsFC data are presented using the maximum observed t-value, maximum p-value under the null distribution and location of the maxima in MNI-space (in a 3 mm-isotropic template). Brain areas were obtained in FSL’s FSLeyes using the Harvard Oxford brain atlas and the Juelich anatomical atlas.

Aside from the pooled analysis, we performed analysis on 3 subgroups: (1) participants who received modafinil then placebo, (2) participants with a large ‘treatment response’ who had an MFI score of <50 at their assessment while on modafinil and (3) treatment non-responders who had an MFI score of ≥60 during their assessment while on modafinil. The MFI score of 50 represents the general population mean^31^ and was chosen to represent a large treatment effect from the baseline inclusion criteria of ≥60.

### Statistical analysis of clinical data

Within-subject differences of the clinical measurements for the primary and secondary analyses were assessed using paired t-tests if assumptions for parametric testing were met, and otherwise the Wilcoxon signed-rank test was used. Statistics presented in the current study are not a formal assessment of the efficacy of modafinil for the overall crossover trial^16^, however we investigated within-subject differences for each analysis individually, to ensure that clinical changes in MFI-20 occurred in the respective analyses. Data are presented with the mean and standard deviations, and assumptions for statistical testing were met unless stated otherwise. Statistical tests on the clinical data were two-tailed and significance level was set at p = 0.05. We performed statistical analyses using the R Statistical Package (version 3.3.2)^32^.

## Results

From the clinical trial, twenty-eight of the thirty-six (77.8%) participants included in MIDAS were eligible for and consented to the MRI-component of the MIDAS study. Participants who underwent the additional MRI scanning had a mean age of 63 years (SD = 14.3), median 3.0 years post-stroke (IQR = 1.5–4.6 years) and ten participants (36%) were female. 15 participants (54.6%) had a stroke in the right hemisphere and 13 participants (46.4%) in the left hemisphere. This cohort had a mean baseline fatigue score on the MFI-20 of 72.3 (SD = 9.24), mean baseline MoCA of 22.1 (SD = 5.21), mean DASS-42 score of 45.3 (SD = 31.61, and a mean baseline SSQoL score of 151.8 (SD = 35.69). Two participants did not partake in baseline scanning due to the possible presence of MRI contraindications, but were confirmed to be eligible for subsequent scanning sessions. One participant could not complete baseline scanning due to claustrophobia. Fourteen participants opted out of one or more scanning sessions (but did complete clinical measurements) during the treatment arms, either as they did not tolerate MRI scanning well, or due to illness. Resting-state data from five participants were excluded due to excessive motion artefacts. Altogether, twenty-one baseline scans, seventeen scans during modafinil treatment, and seventeen scans during placebo were available at each respective time point. Each analysis was based on participants that had an eligible scan available at both time points of interest, and therefore the number of participants in each analysis varies (Fig. 1). We were able to extract the medial, lateral and occipital visual networks, ventral and dorsal default mode network, left and right frontoparietal networks, motor, somatosensory, cerebellar, thalamic and mesolimbic and auditory network. The visual, auditory and cerebellar networks were excluded from further analysis.

### Modafinil versus placebo

*All participants* (*n* = *12*). Self-reported fatigue on the MFI-20 was significantly lower during modafinil treatment compared to during placebo treatment (mean difference −24.9; p < 0.001) and SSQoL was significantly higher (mean difference 28.9; p = 0.015; Table 1). rsFC was significantly higher during modafinil treatment compared to placebo in right hippocampus within the thalamic network (t = 5.681, p = 0.004; Table 2).

**Table 1:** Clinical assessment outcomes.

	Modafinil	Control condition	Mean difference (SEM)	p-value
**All participants**				
*Modafinil versus placebo (n* = *12)*		**Placebo**		
MFI-20	47.0 (10.15)	71.9 (13.52)	−24.9 (5.41)	<0.001
SSQoL	188.6 (3.71)	156.7 (30.01)	28.9 (10.05)	0.015
DASS-42	29.0 (22.3)	35.3 (25.9)	−6.3 (6.04)	0.323
*Modafinil then placebo (n* = *7)*		**Placebo**		
MFI-20	43.1 (10.57)	74.3 (14.44)	−31.1 (7.10)	0.005
SSQoL	187.6 (44.03)	151.7 (25.28)	35.9 (13.68)	0.040
DASS-42	26.0 (21.8)	32.0 (26.6)	−6.0 (8.6)	0.511
**Responders**				
*Modafinil versus placebo (n* = *6)*		**Placebo**		
MFI-20	41.0 (8.47)	76.9 (11.60)	−35.9 (4.65)	<0.001
SSQoL	189.0 (39.95)	145.4 (13.30)	43.6 (13.31)	0.017
DASS-42	22.4 (19.0)	32.9 (18.2)	−10.4 (8.1)	0.246
*Baseline versus modafinil (n* = *8)*		**Baseline**		
MFI-20	42.6 (8.60)	76.3 (9.74)	−33.6 (3.42)	<0.001
SSQoL	180.3 (45.13)	140.6 (22.51)	39.6 (12.07)	0.013
DASS-42	21.0 (18.9)	45.6 (32.1)	−24.6 (6.4)	0.006
**Non-responders**				
*Modafinil versus placebo (n* = *5)*		**Placebo**		
MFI-20	55.4 (4.98)	65.0 (14.09)	−9.6 (6.90)	0.236
SSQoL	188.0 (30.30)	179.6 (36.89)	8.4 (10.69)	0.476
DASS-42	38.2 (25.4)	38.6 (36.3)	−0.4 (9.4)	0.625
*Baseline versus modafinil (n* = *7)*		**Baseline**		
MFI-20	56.0 (4.69)	74.6 (8.30)	−18.6 (3.61)	0.002
SSQoL	185.1 (27.44)	149.6 (55.47)	35.6 (11.56)	0.022
DASS-42	39.7 (26.7)	56.0 (40.7)	−16.29 (7.7)	0.079

MFI-20: Multi-dimensional Fatigue Inventory-20, MoCA: Montreal Cognitive Assessment, SSQoL: Stroke Specific Quality of Life, DASS-42: Depression, anxiety and stress scale-42.

**Table 2:** Locations (MNI-coordinates) and maxima of significant clusters indicating significantly higher functional connectivity during modafinil treatment compared to the control conditions.

Network	Location	T_max_*	P_max_*	Cluster peak			N voxels (volume)
				X	Y	Z	
***All participants: Modafinil versus placebo*** **(n** = **12)**							
Thalamic	R hippocampus	5.681	0.004	24	−30	−6	11 (0.288 mL)
***Responders: Baseline versus modafinil*** **(n** = **8)**							
Ventral default mode	L premotor cortex	4.303	0.035	−3	−6	57	4 (0.105 mL)
***Non-responders: Baseline and modafinil*** **(n** = **7)**							
Thalamic	L thalamus	4.569	0.016	18	−33	18	31 (0.812 mL)

*T-statistics and p-values obtained from permutation testing with threshold-free cluster enhancement.

*Modafinil followed by placebo* (*n* = *7*). In participants who were taken off active therapy as part of the crossover study design, there was a significant increase in the self-reported fatigue on the MFI-20 during placebo treatment (mean difference −31.1; p = 0.005) and a significant decrease in SSQoL (mean difference 35.9; p = 0.040). After the wash-out period of 1 week, fatigue scores within this group were significantly increased (mean difference = 21.9, SD = 22.4, p = 0.003). The removal of modafinil was associated with lower rsFC during placebo within the left inferior parietal lobule in the left frontoparietal network (t = 4.929, p = 0.023), the primary somatosensory cortex within the somatosensory network (t = 4.526, p = 0.009), and the left temporal pole in the mesolimbic system (t = 4.530, p = 0.016).

*Responders* (*n* = *6*). In the subset of patients with an MFI score lower than 50 during modafinil treatment, MFI-20 scores were significantly lower during modafinil treatment compared to during placebo (mean difference −35.9; p < 0.001) and SSQoL was significantly higher (mean difference 43.6; p = 0.017). A significant decrease in rsFC was observed in this group in the somatosensory network (right superior temporal gyrus [t = 4.851; p = 0.016] and right primary somatosensory cortex [t = 3.852; p = 0.031]), in the left frontoparietal network (left inferior parietal lobule [t = 4.962; p = 0.047]), and in the mesolimbic network (left brainstem [t = 4.389; p = 0.047]).

**Figure 2:**
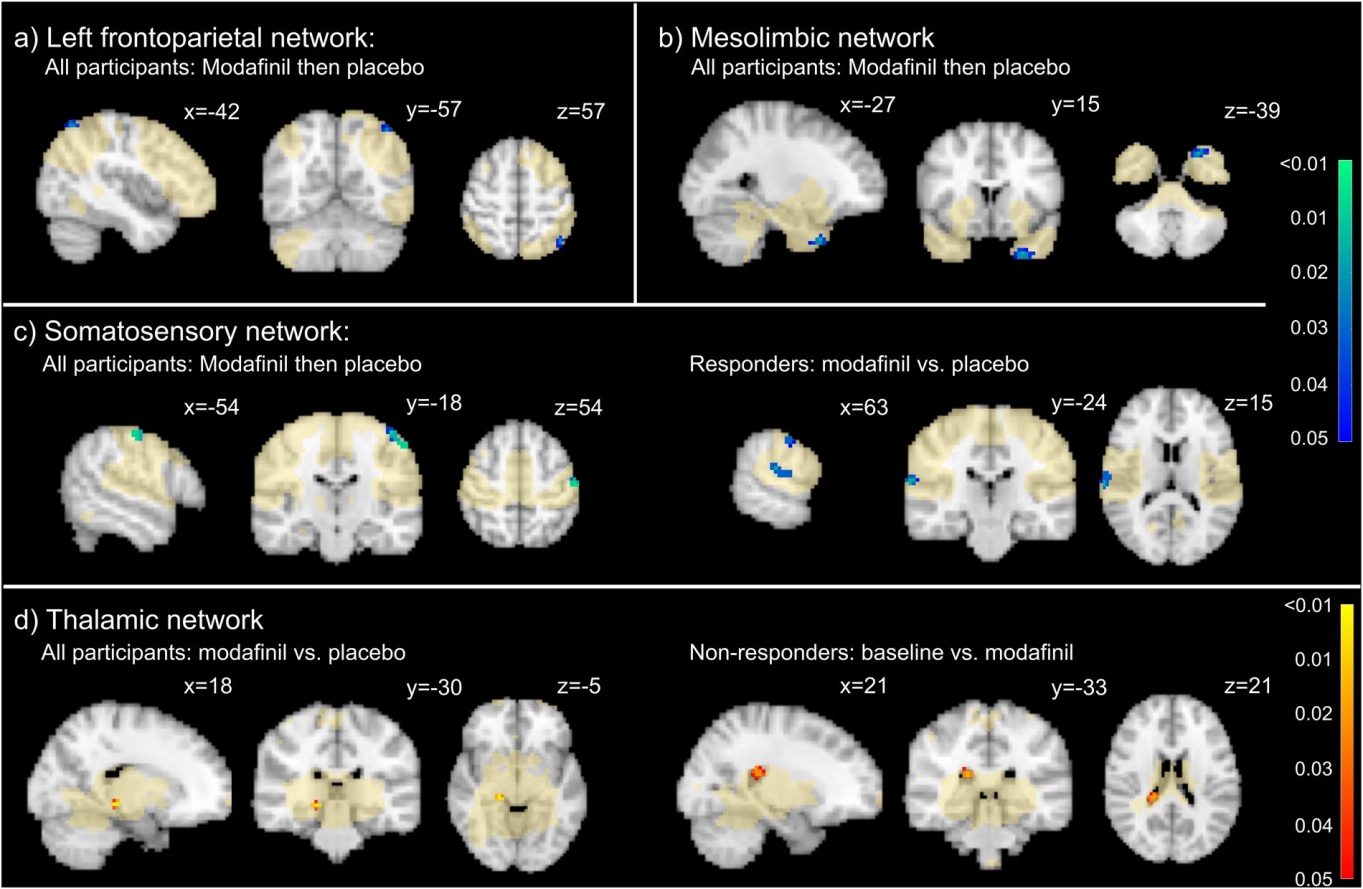
Resting-state functional connectivity (rsFC) changes during modafinil treatment. In the participants who received modafinil first and subsequently placebo (**a**) we observed a significant decrease of rsFC in the left inferior parietal lobe within the left frontoparietal network. Additionally, in participants who received modafinil first, we observed a decrease in rsFC in the left temporal pole of the mesolimbic network (**b**) and in the left somatosensory cortex of the somatosensory network (**c**). In the same network, we observed a significant decrease in rsFC within the responder group in the right superior temporal gyrus and the right somatosensory cortex. Lastly, when all participants were analysed, we observed a significant increase in rsFC between modafinil and placebo treatment within the thalamic in the right hippocampus and in the non-responders we observed a significant increase in the right thalamus between baseline and modafinil treatment (**d**). Red-yellow colours indicate significant p-values (obtained from permutation testing with threshold-free cluster enhancement) for higher rsFC during modafinil treatment, where blue-green colours indicate lower rsFC.

*Non-responders (n* = *5)*. MFI scores did not significantly differ during modafinil treatment and baseline (mean difference −9.6, p = 0.236), nor did SSQoL scores (mean difference 8.4; p = 0.476). Within the ventral default mode network, connectivity within the left posterior cingulate gyrus was significantly lower during modafinil treatment compared to placebo (t = 6.676; p = 0.031; Table 3 and Fig. 2).

**Table 3:** Locations (MNI-coordinates) and maxima of significant clusters indicating significantly lower functional connectivity during modafinil treatment compared to the control conditions.

Network	Location	T_max_*	P_max_*	Cluster peak			N voxels (volume)
				X	Y	Z	
***All participants: Modafinil then placebo (n*** = ***7)***							
Left frontoparietal	L Inferior parietal lobule	4.929	0.023	−42	−60	57	13 (0.340 mL)
Somatosensory	L Primary somatosensory cortex	4.526	0.009	−54	−20	54	32 (0.838 mL)
Mesolimbic	L Temporal pole	4.530	0.016	−27	15	−39	35 (0.917 mL)
***Responders: Modafinil and placebo*** **(n** = **6)**							
Left frontoparietal	L Inferior parietal lobule	4.962	0.047	−42	−60	57	13 (0.340 mL)
Somatosensory	R Superior temporal gyrus	4.851	0.016	−66	−24	15	36 (0.942 mL)
	R Primary somatosensory cortex	3.825	0.031	63	−15	39	6 (0.158 mL)
Mesolimbic	L Brainstem	4.389	0.047	−3	−21	−9	7 (0.183 mL)
***Non-responders: Modafinil and placebo*** **(n** = **5)**							
Ventral default mode	L Posterior cingulate gyrus	6.676	0.031	−3	−24	45	8 (0.210 mL)

^*^T-statistics and p-values obtained from permutation testing with threshold-free cluster enhancement.

### Baseline versus modafinil

*Responders (n* = *8)*. MFI-20 scores were significantly reduced by 33.6 points (p < 0.001) during modafinil treatment compared to baseline. Additionally, SSQoL was significantly increased by 39.6 points during modafinil treatment (p = 0.043), and DASS-42 score was lower by 24.6 points (p = 0.006). This coincided with a significant increase of rsFC within the left premotor cortex in the ventral default mode network (t = 4.303; p = 0.035).

*Non-responders (n* = *7)*. MFI-scores were significantly lower during modafinil therapy compared to baseline (mean difference 18.6, p = 0.002), and SSQoL was significantly higher (mean difference 35.6; p = 0.022). A significant increase of rsFC was observed in the left thalamus within the thalamic network (t = 4.569; p = 0.016; Table 2 and Fig. 2).

## Discussion

In stroke survivors, we have shown that significant changes in resting-state functional connectivity (rsFC) of the left frontoparietal, somatosensory, mesolimbic and thalamic networks occur during modafinil treatment. This coincided with an alleviation of self-reported fatigue and an increase in quality of life. We hypothesized that modafinil would increase rsFC in attentional and cognitive networks due to the mechanisms of modafinil, however, our current findings suggest that modafinil treatment increases in the thalamic network and decreases in the frontoparietal, somatosensory and mesolimbic networks.

The inferior parietal lobe is a brain area associated with sensory integration relating to visuospatial information^33^. When healthy subjects performed a visuo-sensorimotor task after a single-dose (200 mg) of modafinil, task-induced deactivation decreased in the left inferior parietal lobule, indicating that these findings may relate to modafinil administration rather than fatigue-resolution^19^. Dopamine has shown to regulate rsFC of the frontoparietal network with the default mode network^34,35^, and therefore we hypothesize that our findings may be a reflection of altered rsFC in response to modafinil-induced dopaminergic changes. Although modafinil is thought to primarily upregulate orexin, dopamine and its receptors have been shown to be important for the effect of modafinil^36^. Furthermore, orexin is involved in the regulation of dopamine^37,38^, which indicates that our findings may reflect resting-state changes due to dopamine changes which are in turn driven by modafinil-induced upregulation of orexin.

We additionally observed decreased rsFC in the temporal pole within the mesolimbic network. The temporal pole has been suggested to be involved in social and emotional processing as well as semantic memory processing^39^. The mesolimbic network contains the mesolimbic pathway, which is one of the major dopaminergic pathways in the brain associated with the reward response and motivation^40^. Qualitative data showed that motivation is a major theme in post-stroke fatigue^41^, and data from the main MIDAS trial indicated a significant improvement in the motivation sub-domain of the MFI-20^16^. Given that modafinil has shown to influence dopamine levels in the brain^42^, these results may be a reflection modafinil-induced upregulation of dopamine in the mesolimbic system, which may be associated with increase in reported motivation.

We also showed a decrease of rsFC in the primary somatosensory cortex of the somatosensory network. Using independent component analysis, we were able to construct a distinct motor and somatosensory network, rather than a combined sensorimotor network. This indicates that our findings reflect somatosensory changes, rather than functional changes in the motor network. Limited data is available on the role of somatosensory processing in mental fatigue, however, a study in healthy participants indicated that somatosensory-evoked potentials measured using electroencephalography (EEG) were modulated after administration of modafinil and hypothesized that these changes related to alertness and arousal of the somatosensory system^43^. The changes in the inferior parietal lobule and the primary somatosensory cortex suggest a possible role of altered sensory processing in post-stroke fatigue; however, as we did not measure sensory processing across multiple domains, this relationship needs further exploration.

In this study we report alterations of rsFC in networks that can potentially be influenced by dopamine, however we cannot definitively state that modafinil-induced dopamine modulation is responsible for the network changes, nor for the resolution of fatigue. The mesolimbic network, associated with the dopaminergic mesolimbic pathway, has been previously implicated in MS-related fatigue^44,45^. Dysregulation of dopamine was shown in not only MS-related fatigue^46^, but also in cancer-related fatigue^47^, indicating a possible overlap in the mechanisms of fatigue disorders. However, it was shown that dopamine is associated with increased rsFC in the somatosensory network^48^, indicating that dopamine may not be the underlying substrate of our findings, where we showed a decrease in rsFC in this network.

In contrast, dopamine has shown to be associated with decreased rsFC of the default mode network, indicating differential effects of dopamine on specific networks. Additionally, decreases of rsFC have been shown in relation to the serotonin signalling pathway^48^, indicating that modafinil-associated fatigue reductions may be related to serotonin, rather than dopamine.

Studies have shown that post-stroke fatigue is associated with the presence of inflammatory markers C-reactive protein and interferon-α^12^. Modafinil has shown to suppress the expression of proinflammatory cytokines^49^, and therefore modafinil may reduce neuro-inflammation and thereby reduce fatigue. Inflammatory markers in turn influence the availability of serotonin and dopamine and could thereby alter rsFC^50^. Thus, the effects of dopamine and serotonin on rsFC need to be further explored to improve our understanding of their role in pathological fatigue.

We mainly observed decreases in rsFC, which was in contrast to our a-priori hypothesis. Studies on rsFC after modafinil administration are scarce, however, those available indicate an increase of connectivity in areas such as the frontal cortex^51^, the posterior insula^52^, and the anterior cingulate cortex^20^. Our findings showed a decrease in rsFC in several brain networks, which may be indicative of post-stroke fatigue related hyper-connectivity, which normalizes after modafinil administration. Increases of rsFC in the posterior cingulate cortex, primary and supplementary motor cortex have been reported in patients with multiple sclerosis-related fatigue compared to non-fatigued MS patients^53^. The question of whether increased rsFC patterns occur in post-stroke fatigue would need to be addressed in a future study comparing brain networks in fatigued stroke survivors to those of non-fatigued stroke survivors.

Ischaemic stroke is likely to cause changes in neurovascular coupling, cerebral blood flow, and cerebral blood volume, which are factors that influence the blood-oxygen level dependent signal on which resting-state fMRI are based^54^. This could lead to increased noise within the signal, which could theoretically obscure any of the effects of interest. However, in the current study we included only within-subject comparisons and worked under the assumption that these factors would show minimal change in the time course of the study, as patients were more than three months post-stroke and would therefore have a marginal impact on our findings. We had sufficient participants to extract the resting-state networks of interest, indicating that there was enough shared variance between the participants. However, sample sizes were small (ranging from five to twelve), as this study was limited by the number of participants that were eligible for the neuroimaging component of the study and therefore we may have had insufficient power to show further smaller effects. Additionally, we applied permutation tests to assess differences, which is a more suitable method for smaller sample sizes. Currently, no formal methods for the calculation of sample sizes are available for resting-state fMRI studies; however, in studies with stroke populations where effectiveness of treatment and concurrent rsFC changes have been investigated, sample sizes fluctuate between 8 to 12 participants^55–57^.

A further limitation of this study was that the imaging assessor was not blinded to the study outcomes and the group allocation. However, data acquisition was performed by a blinded assessor. Since this study was not a formal investigation on the efficacy of modafinil, we believe that this does not impact our current findings. Furthermore, we selected participants that were eligible (MRI safety) and consented to the imaging arm of this study. There were no significant differences in fatigue scores between those participants that were included in the imaging study and those who only participated in the main MIDAS trial^58^.

## Conclusions

Modafinil treatment-associated alleviation of post-stroke fatigue coincided with altered rsFC in the left frontoparietal network, somatosensory network, thalamic and mesolimbic network. We cannot make inferences regarding causal relationships between fatigue and rsFC, but the neuroimaging data does indicate a potential role for sensory processing and motivational deficits in post-stroke fatigue. Modafinil may aid in the alleviation of these deficits, and thereby reduce perceived fatigue. Changes in networks susceptible to dopamine expression indicate a possible role for this neurotransmitter in post-stroke fatigue. Future studies are needed to identify differences in sensory processing and dopamine levels between stroke survivors and their non-fatigued peers, and how they relate to rsFC in functional networks.

## Data Availability

The dataset generated during and/or analysed during the current study are available from the corresponding author on reasonable request.
